# Is cardiovascular autonomic control during the day‐to‐night transition dependent on dim light melatonin onset? An exploratory analysis in healthy adults

**DOI:** 10.14814/phy2.71145

**Published:** 2026-10-16

**Authors:** Leandro C. Brito, Megan Jones, Caroline P. Garcês, Steven A. Shea, Saurabh S. Thosar

**Affiliations:** ^1^ Oregon Institute of Occupational Health Sciences Oregon Health & Science University Portland Oregon USA; ^2^ Exercise, Women and Cardiometabolic Health Research Group, Faculty of Physical Education and Physical Therapy Federal University of Uberlandia Uberlandia Brazil; ^3^ School of Nursing Oregon Health & Science University Portland Oregon USA; ^4^ OHSU‐PSU School of Public Health Oregon Health & Science University Portland Oregon USA; ^5^ Knight Cardiovascular Institute Oregon Health & Science University Portland Oregon USA

**Keywords:** cardiovascular autonomic modulation, circadian biology, dim‐light melatonin onset, heart rate variability, sleep

## Abstract

We tested whether dim‐light melatonin onset (DLMO) is time‐aligned with the transition between biological day into night in cardiovascular autonomic control (CV onset time). We studied 10 healthy adults (4 females, age 25 ± SD 2 years) under an ultra‐short semi‐constant routine protocol (<8lux). Participants remained seated for 7 h until 1 h after their habitual bedtime. We collected saliva samples every 30 min and recorded R‐R intervals and beat‐to‐beat blood pressure every hour. DLMO was defined as the interpolated time for melatonin increase over 3 pg/mL. Phase angle was defined as the time difference between DLMO and the midpoint of self‐reported sleep as a measure of the alignment between sleep–wake patterns and biological circadian rhythms. CV onset time was defined using a segmental (piecewise) analysis to identify the inflection point (X_0_ slope) throughout the study. CV autonomic modulation was assessed using indices of heart rate and blood pressure variability. CV onset times for all indices did not differ significantly from DLMO (*p* > 0.05). Mean differences between DLMO and CV onset time were significantly correlated with the phase angle of entrainment (all significant, *r* > +0.75). Our results suggest that CV autonomic transition into biological night was not associated with DLMO, which seems to depend on the phase angle of entrainment.

## 1 INTRODUCTION

Circadian modulation of the body's physiological state prepares the body to respond to anticipated daily changes in behaviors and the environment (Refinetti, 2020). The synchronization of the circadian system with daily behavioral cycles and environmental changes is essential for optimal physiological adjustment during transitions such as day‐to‐night and vice versa. Cardiovascular autonomic control is greatly affected during the transition from day to night, as demonstrated in controlled circadian studies. For instance, resting cardiac parasympathetic activity is low and starts increasing before the biological night (Scheer et al., 2004), while norepinephrine (i.e., a marker of the sympathetic nervous system) has its highest values during the biological day (~5 p.m.), when norepinephrine starts decreasing to reach its lowest value during the biological night (Scheer et al., 2010). Along these lines, the endogenous increase in melatonin, which occurs on average 2 h before habitual sleep in healthy young adults (Burgess et al., 2003), has been hypothesized to be a potential mechanism responsible for the transition in cardiovascular autonomic control to the resting nighttime mode (Burgess et al., 1997). However, this hypothesis was rejected when melatonin levels were manipulated around bedtime without affecting cardiac autonomic modulation (Burgess et al., 2001).

Melatonin (*N‐acetyl‐5methoxytryptamine*) is a hormone synthesized by the pineal gland at night when under dim light conditions as controlled by the endogenous circadian system (Czeisler et al., 1999). Plasma melatonin levels normally follow a circadian rhythm with an increase in the evening, which marks the start of the dark phase (Czeisler et al., 1999). Aside from its circadian‐regulatory effects, melatonin directly impacts cardiovascular functions (Covassin & Somers, 2023), and low melatonin levels are observed in people with established cardiovascular disease (Brugger et al., 1995; Sun et al., 2024). Among several cardioprotective effects of melatonin, its action on cardiovascular autonomic regulation has been reported following both acute and multi‐week melatonin supplementation. Notably, 3 months of melatonin supplementation increased cardiac parasympathetic balance during sleep in pinealectomized patients by increasing vagal‐dominated heart rate variability (HRV) indices, such as the absolute high‐frequency band of HRV and the square root of the mean of the sum of the squares of differences between adjacent norma‐to‐normal (NN) intervals (RMSSD) (Campos et al., 2020). The same effect was no longer observed 3 months after discontinuing melatonin supplementation, suggesting that the effects can be sustained over long periods and yet still reversed (Campos et al., 2020). In young healthy adults, a single ingestion of 2–3 mg melatonin tablet attenuated the muscle sympathetic nerve response to orthostatic stress and increased cardiac parasympathetic modulation when administered in the morning (~10 a.m.) (Nishiyama et al., 2001). However, exogenous melatonin administered at the estimated dim light melatonin onset (DLMO) [i.e., when levels of melatonin normally increase] did not promote a change in the autonomic control of heart rate (HR) compared to placebo (Burgess et al., 2001). In addition, the same study found no changes in autonomic control of HR after melatonin suppression by exposing participants to bright light (>3000 lux) (Burgess et al., 2001). However, it is unknown whether endogenous melatonin facilitates the autonomic nervous system's transition to a quiescent state, marking the biological night. Thus, the current study primarily tested whether cardiac parasympathetic modulation, assessed by RMSSD from biological day to biological night is associated with the endogenous increase in melatonin identified by DLMO, as well as secondary CV metrics such as heart rate, BP, and related CV autonomic indices. To test this hypothesis, participants were assessed in an ultra‐short semi‐constant routine protocol while remaining seated in a comfortable chair for 7 h under dim‐light (<8 lux) that ended 1 h after participants' habitual sleep time.

## 2 METHODS

### 2.1 Participants

Young healthy adults aged 20–39 years were recruited through posted flyers, word of mouth, and volunteer databases. Participants were initially screened over the phone and considered eligible if they reported no chronic medical conditions and no use of prescribed medications. In addition, eligible participants had no history of night‐shift work or tobacco use within the prior 12 months, did not use marijuana or illicit drugs, and were not pregnant, as confirmed by urinalysis in female participants. This study was approved by the Institutional Review Board (IRB 24564) for human subject protection at Oregon Health & Science University (OHSU), and the procedures were conducted in accordance with the Declaration of Helsinki. All participants provided written informed consent prior to participation.

### 2.2 Study design

#### 2.2.1 At‐home monitoring

Prior to the in‐lab visit, participants selected an eight‐hour in‐bed schedule to regularize their sleep–wake patterns and circadian rhythms for 7 days. Every day, participants called into a time‐stamped mailbox to indicate when they went to bed intending to sleep and when they woke up to confirm their adherence. They were also asked to complete a sleep diary with questions about sleep and wake times, as well as sleep continuity (e.g., how long it took to fall asleep and the number of nighttime awakenings). If a participant missed a time‐stamped call‐in, a staff member contacted them to check in and remind them to keep track of their calls. Participants were re‐informed that their participation in the study would be terminated if they did not make time‐stamped calls. No participant was excluded from this study. Before the in‐lab visit, participants were asked to abstain from vigorous exercise for 48 h, and from caffeine, alcohol, and nonprescription drugs, vitamins, and supplements for 24 h.

#### 2.2.2 In‐lab visit

Participants arrived at the lab 8 h prior to their selected bedtime and stayed for 1 h after that bedtime. Pregnancy testing was conducted for all female participants during screening visits (Pregnancy tests (HCG), Easy@Home, Burr Ridge, IL). Nicotine (NicAlert; Nymox Corporation, NJ), recreational and opiate drugs (Drugsmart 12 panel cup; Speares Medical, Inc., SC) were also tested during screening for all participants. Female participants were studied during the early follicular phase of their menstrual cycle, except for two participants using hormonal birth control when the period of the cycle was not controlled. Afterward, resting blood pressure (BP) was measured to confirm that it fell within eligibility requirements, with systolic and diastolic BP < 130 mmHg and < 80 mmHg, respectively. We measured BP using an oscillometric device (Dinamap, GE Medical Systems, Milwaukee, WI) after 10 min of seated rest in triplicate on each arm, with a 5 min interval between measurements (Whelton et al., 2018). BP values were averaged. Height and weight were measured to ensure BMI fell between 18.5 and 29.99 kg/m^2^.

After confirming eligibility, lights were dimmed (<8 lux), and participants remained seated for the next 7 h, except for restroom use, which was managed to end at least 10 min prior to melatonin or autonomic measurements in an ultra‐short semi‐constant protocol (Figure 1). Notably, the restroom was in the same laboratory suite and had the same light levels (<8 lux). Every half hour, we collected a saliva sample using a Salivette cotton swab (Sarstedt, Inc., NC). Every hour, we recorded 10 min of beat‐by‐beat blood pressure and ECG data to assess the cardiovascular autonomic modulation. During the whole 7 h, participants refrained from any screen contact, such as phones, watches, tablets, or laptops. Participants were allowed to engage in extremely low‐effort activities such as reading a book, writing, book puzzles, or conversation with research staff, which had to stop 5 min before any measurement. Additionally, water was provided ad libitum, but participants were asked to refrain from drinking for 5 min preceding each Salivette collection. Participants were permitted to eat snacks (e.g., cereal bars or fruit) between autonomic and melatonin measurements, which they finished at least 10 min before the next data assessment, either a resting autonomic or a salivary sample. Participants were required to brush their teeth immediately after a snack to avoid contamination of the saliva sample with food.

**FIGURE 1:**
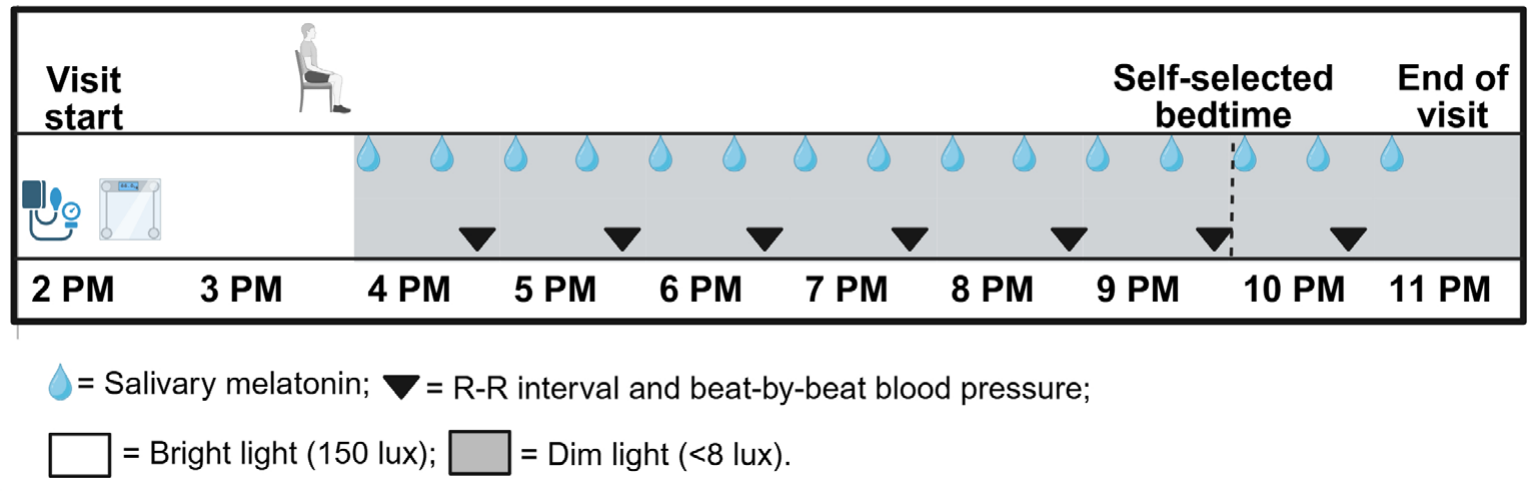
Ultra‐short **s**emi‐constant study protocol. After confirming study eligibility. Participants remained seated in a comfortable chair for the next 7 h. Data collection started 6 h before the self‐selected bedtime (vertical dashed line), which participants had followed for 7 days before the visit. Light blue drops represent salivary samples collected every 30 min. Black triangles represent 10‐min recordings of resting ECG and beat‐to‐beat blood pressure, obtained every 1 h. The white shaded area represents room lighting at 150 lux; and the gray shaded area represents dim light wake periods below 8 lux.

### 2.3 Measurements

#### 2.3.1 Circadian phase and phase angle

Saliva samples were collected using Salivette cotton swabs (Sarstedt, Inc., NC) and immediately placed on ice to minimize degradation. Samples were then centrifuged (1000 g for 2 min) and stored in a − 80°C freezer until analysis. Salivary melatonin concentrations were quantified as previously described (Thosar et al., 2019). DLMO was defined as the linearly interpolated time at which melatonin levels exceeded 3 pg/mL (Benloucif et al., 2008). Melatonin was measured every 30 min across a 7‐h period, including 1 h past each participant's habitual bedtime to ensure that DLMO was captured. Phase angle of entrainment between habitual sleep–wake patterns and the endogenous circadian system was calculated as the time difference between DLMO and the average diary‐reported sleep midpoint from 7 days of at‐home data (Nota et al., 2025).

#### 2.3.2 Cardiac R‐R interval, blood pressure, and cardiovascular autonomic modulation

The 10‐min autonomic measurements consisted of continuous ECG and beat‐by‐beat blood pressure recordings. A standard three‐lead ECG was used to measure the cardiac R‐R interval using a data acquisition system (LabChart version 8.0; ADInstruments). Beat‐by‐beat blood pressure was measured via photoplethysmography using a finger cuff positioned on the middle finger of the participant's nondominant hand (NOVA Finometer, Finapres Medical Systems, Amsterdam, The Netherlands), which has been previously validated against intra‐arterial measurements (Parati et al., 1989). Cardiac R‐R intervals and BP averages were calculated over the 10‐min period.

Cardiac R‐R interval and BP signals were sampled at 1000 Hz/channel and acquired using LabChart version 8.0 (AD instruments). After visual inspection to remove artifacts and ectopic beats, data were imported into an analysis software (CardioSeries v. 2.4, São Paulo, Brazil) of stationary segments while recorded in parallel to a respiratory rate of 12–19 breaths/min using a thoracic piezoelectric belt (Respiratory belt sensor, AD Instrument). We evaluated time‐ and frequency‐domain of HR variability following the recommendations indicated in the task force guidelines about the method (Task Force, 1996). The time‐domain analysis of HR variability was derived from the normal‐to‐normal R‐R interval (NN) by calculating RMSSD ms. For interpretation, RMSSD was used as a marker of cardiac parasympathetic modulation. The frequency‐domain analysis of HR and BP variability was performed using spectral power analysis. Cardiac R‐R intervals and beat‐to‐beat systolic BP were converted into an evenly sampled time series by interpolation at 4 Hz, detrended, and then subjected to frequency‐domain analysis. Power spectral density was calculated using Fast Fourier Transform with a 256‐beat window, applied to successive overlapping segments of the time series. Spectral power was quantified within the standard frequency bands of interest for cardiovascular autonomic modulation. Low‐frequency (LF: 0.04–0.15 Hz) and high‐frequency (HF: 0.15–0.40 Hz) ratio (LFHF_R‐R_) was used as an estimate marker of cardiac sympathovagal balance (Task Force, 1996). Sympathetic vasomotor modulation was assessed by the absolute value of the low‐frequency band of systolic BP variability (LF_SBP_ mmHg^2^) (Pagani et al., 1997).

#### 2.3.3 Determining CV autonomic shift onset time

We determined the moment at which each CV autonomic index metric changed for each participant, considering each time point across the 7‐h visit, plotted in parallel with the clock time at which they were collected. We identified the point at which the slope of each variable shifted in each participant. We determined the inflection point as the *X*_0_ identified through a segmental linear regression (piecewise model) of each CV autonomic index in each participant, as previously used (Studinger et al., 2007), and defined it as the onset time. Segmental linear regression (piecewise model) was plotted using GraphPad Prism version 11 for Windows. This approach was adapted from previous studies using the modified‐Oxford technique to study the cardiac baroreflex sensitivity when it is necessary to identify the inflection point in systolic BP to determine when it starts falling after infusing sodium nitroprusside and rises again after infusing phenylephrine (Brito et al., 2024; Sabino‐Carvalho et al., 2020). Thus, the segmental linear regression (piecewise model) approach detects the time at which an autonomic variable shifts its pattern, regardless of whether the change is sustained upward or downward. An experienced researcher (L.C.B.) verified each segmental linear regression to ensure the selected point corresponded to a physiologically meaningful transition rather than transient noise caused by an isolated outlier. We defined an outlier as a value more than 3 SDs from the mean. Notably, we identified one RMSSD data outlier from one participant and removed that data point. Thus, one participant had their segmental linear regression with 6 rather than 7 data points.

### 2.4 Statistical analysis

This is a novel exploratory analysis from additional unpublished data collected during a separate visit from a circadian study we recently published (Brito et al., 2026). Thus, the study was not powered to answer these particular questions.

Data normality was assessed using the Shapiro–Wilk test.

Cardiac R‐R interval, BP, and CV autonomic onset times were extracted for each index, evaluated and compared with DLMO. Paired *t*‐tests were used for mean comparisons between the shift onset time of CV variables and DLMO. A one‐sample *t*‐test was employed to determine when the onset times of CV variables began to differ statistically from DLMO. To test this, the mean difference between the onset time of the respective CV variable and DLMO was sequentially compared with absolute decimal increments representing an interval of 10 min (i.e., versus 0.1, 0.2, 0.3, etc.) until a *p*‐value <0.05 was reached, which was arbitrarily designated as the threshold of significance (TS). Notably, the one‐sample *t*‐test was employed for negative and positive values. Finally, we calculated Pearson's correlation coefficient between the sleep patterns (i.e., diary‐reported bedtime and midpoint sleep) and the phase angle (i.e., as a marker of circadian alignment), using the difference between the onset time of each CV variable and DLMO. The multiple correlations were corrected using Benjamini‐Hochberg False Discovery Rate method (Haynes, 2013). All statistical analyses were performed using SPSS for Windows, v.29, IBM Corp., Armonk, NY, USA. Significance was set at alpha = 0.05.

## 3 RESULTS

We studied 10 healthy young adults, and their characteristics are shown in Table 1. During the 7 days at home, the average self‐selected bedtime was 10:47 p.m. ± 00:28 and wake time was 6:58 a.m. ± 00:33 hh: mm (Mean ± SD), while bedtime variability was 6 ± 9 min and sleep midpoint variability was 5 ± 7 min. The average DLMO was 8:26 p.m. ± 00:57 hh: mm. For the segmental linear regression (piecewise model) used to determine the CV shift onset time, all goodness‐of‐fit *R* ^*2*^ values were equal to or higher than 0.6. Average and minimum‐maximum variations for each variable were, respectively: 0.85 and 0.61–0.99, for R‐R interval, 0.82 and 061–0.98 for RMSSD, 0.82 and 0.62–0.96 for LF/HF_R‐R_, 0.75 and 0.60–0.95 for systolic BP, and 0.73 and 0.63–0.88 for low‐frequency systolic BP.

**TABLE 1:** Characteristics of the participants.

Variables	
Male/Female (*n*)	6/4
Race	
White (*n*)	6
Black (*n*)	1
American Indian (*n*)	1
More than one race (*n*)	1
Unknown (*n*)	1
Ethnicity	
Hispanic or Latino	1
Non‐Hispanic or Non‐Latino	9
Age (years)	25 ± 2
Height (cm)	173 ± 9
Body mass (kg)	75.8 ± 7.5
Body mass index (kg/m^2^)	25.4 ± 2.3
Systolic blood pressure (mmHg)	116 ± 7
Diastolic blood pressure (mmHg)	66 ± 6
Heart rate (bpm)	65 ± 7

*Note*: Values are shown as mean ± standard deviation or *n* (number of participants). Blood pressures were measured in triplicate in the office during screening visits. Hour: minutes (hh:mm).

Figure 2 shows the mean differences between DLMO and the onset time of each CV variable, which were not statistically different after mean comparisons, and their positive and negative threshold of significance, respectively. R‐R interval (34 ± 86 min, *p* = 0.244, with −24 to 96 min of TS, and Cohen's *d* = 0.39), RMSSD (−25 ± 101 min, *p* = 0.450, with −96 to 45 min of TS, and Cohen's *d* = 0.25), LF/HF_R‐R_ (−33 ± 87 min, *p* = 0.269, with −90 to 24 min of TS, and Coehn's *d* = 0.37), systolic BP (18 ± 92 min, *p* = 0.537, with −42 to 84 min of TS, and Cohen's *d* = 0.20), and low‐frequency systolic BP (12 ± 86 min, *p* = 0.669, with −54 to 72 min of TS, and Cohen's *d* = 0.14).

**FIGURE 2:**
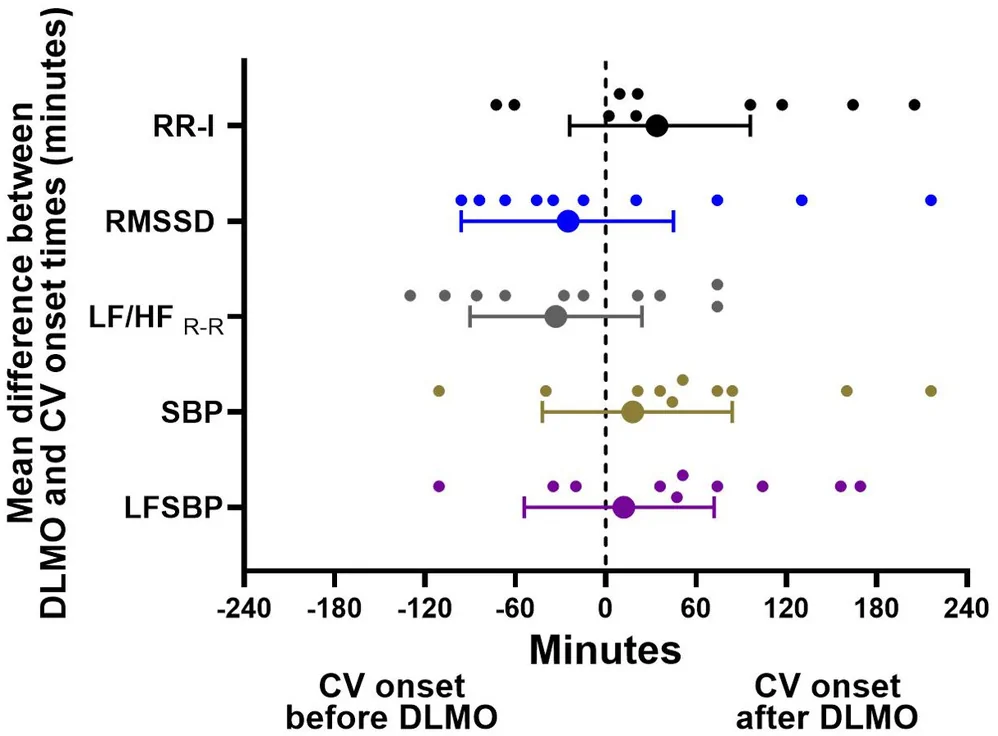
Mean difference between dim‐light melatonin onset (DLMO) and the onset time of each cardiovascular (CV) index. Mean values are plotted with their respective thresholds of significance for each index, which were determined by employing one‐sample *t*‐tests sequentially compared with absolute decimal increments representing an interval of 10 min (i.e., versus 0.1, 0.2, 0.3, etc.) until a *p*‐value <0.05. Negative thresholds of significance mean CV onset occurred before DLMO while positive values mean after DLMO. R‐R interval (RR‐I); Root of the mean of the sum of the squares of differences between adjacent NN intervals (RMSSD); Low‐frequency and high‐frequency ratio of RR‐I variability (LF/HF_R‐R_); systolic blood pressure (SBP); and Low‐frequency SBP variability (LFSBP).

Coefficients of correlation with original *p*‐values and adjusted *p*‐values for multiple correlations are shown in Table 2, while only scatter plot graphs of statistically significant correlations after adjustments are shown in Figure 3. Phase angle showed a statistically significant positive correlation with the mean difference between DLMO and the onset time of CV variables, except R‐R interval. Diary‐reported average bedtime and midpoint sleep were not significantly associated with the mean difference between DLMO and the onset time of any CV variable (all *p* > 0.05), except for R‐R interval. For interpretation, positive mean differences between the onset time of CV variables and DLMO refer to an onset time of CV variable that occurred after DLMO (i.e., blue arrows in Figure 3 graphs), while negative values in the same graph refer to an onset time of CV variables that occurred before DLMO (i.e., red arrows in Figure 3 graphs).

**TABLE 2:** Person correlation *p*‐values and adjusted *p*‐values using Benjamini‐Hochberg's false discovery rate correction.

Dependent variables: Mean difference between DLMO and	Independent variables	*r*	*p*‐value	Adjusted *p*‐value
R‐Ri onset time	Phase angle	+0.586	0.075	0.160
	Bedtime	**+0.836**	0.003	**0.013**
	Midpoint sleep	**+0.818**	0.004	**0.014**
RMSSD onset time	Phase angle	**+0.861**	0.001	**0.010**
	Bedtime	+0.197	0.586	0.627
	Midpoint sleep	+0.036	0.922	0.922
LF/HF_R‐R_ onset time	Phase angle	**+0.781**	0.008	**0.023**
	Bedtime	+0.414	0.234	0.390
	Midpoint sleep	+0.286	0.423	0.529
Systolic BP onset time	Phase angle	**+0.763**	0.010	**0.026**
	Bedtime	+0.310	0.383	0.523
	Midpoint sleep	+0.238	0.509	0.922
LF systolic BP onset time	Phase angle	**+0.888**	<0.001	**0.009**
	Bedtime	+0.444	0.199	0.373
	Midpoint sleep	+0.328	0.356	0.533

*Note*: Bold adjusted *p* values are significant.

Abbreviations: BP, blood pressure; DLMO, dim‐light melatonin onset; LF/HF_R‐R_, low‐to high‐frequency bands ratio of R‐R interval; RMSSD, root mean square of successive differences.

**FIGURE 3:**
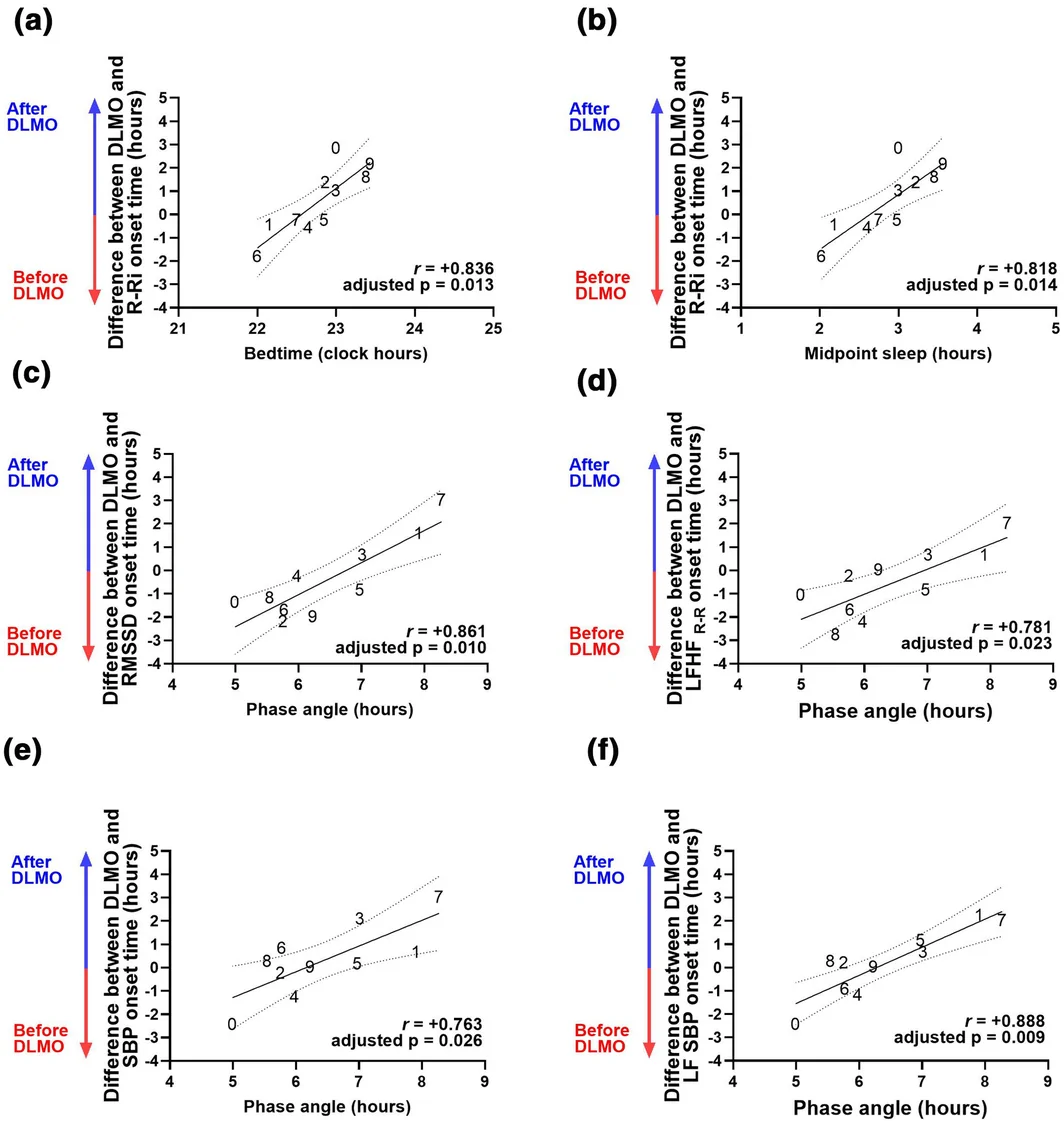
Pearson correlation between the difference of dim‐light melatonin onset (DLMO) and shift onset time of R‐R interval (R‐Ri) and bedtime (panel a); R‐Ri and mispoint sleep (panel b); square root of the mean of the sum of the squares of differences between adjacent NN intervals (RMSSD) and phase angle (panel c); low to high‐frequency ratio of R‐Ri variability (LFHFR‐Ri) and phase angle (panel d); systolic blood pressure (SBP) and phase angle (panel e); and low‐frequency of SBP variability (LF SBP) and phase angle (panel f). Considering an adjusted *p*‐value using Benjamini‐Hochberg's false discovery rate correction. Numbers represent the same participant in every scatter plot.

## 4 DISCUSSION

This study aimed to investigate whether the transition in CV autonomic modulation from biological day to biological night depended on the endogenous increase in melatonin identified by DLMO while controlling for body posture, sleep, and light, while allowing participants to drink water and eat ad libitum in an ultra‐short semi‐constant environment. Our data do not support our hypothesis, and the timing of the transition to biological night for cardiac parasympathetic modulation assessed by RMSSD, as well as the secondary CV variables investigated, did not depend on the endogenous increase in melatonin. Otherwise, the onset time of each CV variable investigated occurred around DLMO, with some participants having it before DLMO and others after; this varied by the index assessed (Figure 2). A novelty in our findings was that the individual variability was strongly and positively associated with phase angle of entrainment. Thus, people with a wider phase angle had their CV onset after DLMO, whereas those with a narrower phase angle had their CV transition before DLMO (Figure 3).

The concept of an increase in endogenous melatonin influencing subsequent changes in CV autonomic modulation has not been previously investigated. Previous studies showed no influence of endogenous melatonin preceding bedtime on cardiac autonomic modulation (Burgess et al., 1997, 2001). One of these studies experimentally suppressed melatonin by using bright light (>3000 lux) and compared it to melatonin levels during dim light (<10 lux) (Burgess et al., 2001). In that study, there was a decrease in core body temperature and an increase in peripheral (foot) temperature, but the indices used to assess cardiac parasympathetic and sympathetic modulation did not change with melatonin suppression (Burgess et al., 2001). Similarly, the current study found that neither parasympathetic nor sympathetic indices of cardiac autonomic modulation nor sympathetic vasomotor modulation depended on endogenous melatonin.

Although CV onset did not depend on increased endogenous melatonin, it still occurred around DLMO, suggesting that the endogenous circadian system may contribute to autonomic regulation of CV as it transitions into a state of biological night, especially in its alignment with behaviors (e.g., sleeping). Our findings suggest that participants with a narrower phase angle (i.e., a shorter time between DLMO and midpoint sleep) consistently had CV onset before DLMO, whereas this did not occur in those with a wider phase angle (see Figure 3). Notably, none of our subjects had worked night shifts in the past 12 months or longer, subjects reported no sleep disturbance, and regularized their time in bed for 7 days before the in‐lab visit. The anticipation of CV onset in participants with a narrower phase angle suggests a physiological readiness to anticipate transitions or environmental changes, compared with physiology simply adjusting to environmental changes or behaviors via negative feedback control. Changes in resting CV variables, especially those under circadian control are slow. One of the best examples in the CV realm is the increase in heart rate, blood pressure, and circulating cortisol that begins while we are still sleeping, as the body prepares for awakening (Viola et al., 2015). We recognize the observational nature of our study and recommend caution in assuming that a narrower phase angle of entrainment is associated with earlier CV autonomic control preparing for biological night. Nonetheless, our preliminary findings should be interpreted as hypothesis‐generating and encourage future studies to be conducted in larger sample sizes and to experimentally test whether imposed circadian misalignment protocols, such as ‘the slam shift’ (McHill et al., 2024; Morris et al., 2016, 2017), affect the CV autonomic onset. Another aspect that could be tested is whether the alignment between the circadian system and behaviors affects not only physiology on average, but also its efficiency in transitioning between physiological states in behavior, such as resting to activity and awake to asleep, or environmental, such as light to dark and or warm to cold.

Data supporting this theory are observed in studies that found an association between behavioral markers of circadian misalignment and impaired autonomic control of the CV system. For instance, ambulatory data from adolescents (*N* = 360) showed that individuals with a later midpoint sleep had an increased cardiac sympatho‐vagal balance, both awake and asleep (Morales‐Ghinaglia et al., 2025). Similarly, a previous study in healthy adults found that those with higher social jet lag, defined as the difference in sleep timing between workdays and free days (Wittmann et al., 2006), had reduced cardiac parasympathetic modulation during the first 3 h of sleep compared with those with lower jet lag (Sudy et al., 2019). Negative impact on CV function observed in natural models of circadian misalignment has been reproduced in tightly controlled in‐laboratory studies (Morris et al., 2016, 2017). In particular, Morris and colleagues demonstrated that experimentally induced circadian misalignment impairs CV function (Morris et al., 2017). For instance, authors demonstrated that wake systolic BP and 24‐h average diastolic BP increased after a 12‐h inversion of participants' sleep–wake cycle to induce circadian misalignment (Morris et al., 2017). Although heart rate and cardiac parasympathetic modulation were unaffected by this acute circadian misalignment, Morris and colleagues observed a modest increase in heart rate specifically during the sleep opportunity following the misalignment (e.g., 1.8 bpm) (Morris et al., 2017). Thus, it is reasonable to suggest that the alignment between the endogenous circadian system and daily behavioral cycle (i.e., sleep) affects CV autonomic modulation. The current study adds an inter‐individual perspective to the literature by investigating circadian misalignment as a function of preparedness for optimal physiology. Further exploration of the current study's findings could be important for a more comprehensive investigation of the mechanisms connecting how circadian alignment influences the transition of CV autonomic control into the biological night.

The current study benefited from several strengths, but it was not without limitations. The fact that we did not collect melatonin throughout the night limits our findings to the transition from biological day to the biological night. Sex differences have been observed in DLMO, which may occur earlier in females than in males (Cain et al., 2010). Although both female and male participants were investigated, the preliminary analysis characteristics of our small sample size (*n* = 10) limit the statistical power to detect sex differences. However, visual distribution does not suggest a sex effect in our sample. In the scatter plot graph, participants 0 and 1 are females, and participants 7 and 8 are males, and one of each sex is at the widest and narrowest edges of the phase angle (Figure 3c–f). Our sample was restricted to young and healthy adults, and aspects such as aging and other CV risk factors, such as hypertension or obesity, can affect CV autonomic modulation (Dimova et al., 2026; Mancia et al., 1978; Monahan, 2007). We sampled CV autonomic data every hour, which may have affected the accuracy of determining the onset time of the CV autonomic shift. Future studies are encouraged to increase CV autonomic sampling or focus on continuous ECG measurement to assess HR variability only, since our preliminary analysis did not identify any difference between HR or BP variability indices. In the current study, we covered both heart and vasomotor autonomic modulation. To collect truly resting physiology, participants had to remain completely still and awake throughout the recording. This approach made continuous data collection for 7 hours unfeasible in the current study because of the existing participant burden. Thus, we opted for the every‐hour design in our semi‐constant routine protocol, which provided 7 data points, which is more than the minimum 5 data points suggested by Studinger and colleagues for a segmental linear regression (piecewise model) (Studinger et al., 2007). Our results should not be generalized to individuals of different ages and those with comorbidities. However, our highly selective screening process yielded a homogeneous sample of ostensibly healthy participants, enabling us to derive mechanistic insights from our findings.

## 5 CONCLUSIONS

Our exploratory findings do not establish whether the endogenous increase in melatonin determines the transition in CV autonomic modulation into the biological night in ostensibly healthy young adults. In addition, our preliminary results suggest that inter‐individual variation in circadian alignment is strongly associated with the transition in CV autonomic physiology into the biological night. Studies with larger sample sizes and more frequent sampling of autonomic indices and melatonin will increase resolution and help to reconcile the conundrum between endogenous melatonin rise and the shift in CV autonomic modulation.

## AUTHOR CONTRIBUTIONS

L.C.B. and S.S.T. conceived the study; M.J. and L.C.B. conducted the experiments and data analysis; L.C.B., realized the statistical analysis; S.S.T., L.C.B., and S.A.S., obtained the grants and interpreted the results. L.C.B., M.J., and C.P.G. drafted the manuscript. L.C.B., M.J., C.P.G., S.S.T., and S.A.S. edited and revised the manuscript. All authors contributed to the intellectual discussions and approved the final version of the manuscript.

## FUNDING INFORMATION

This work was supported by the Medical Research Foundation of Oregon and American Heart Association Career Development Award 24CDA1267757 (to L.C.B.); National Institutes of Health R01‐HL163232 (to S.S.T.); NIH Grant R35‐HL155681 (to S.A.S.); the Oregon Institute of Occupational Health Sciences at Oregon Health and Science University via funds from the Division of Consumer and Business Services of the State of Oregon (ORS‐656.630); and UL1‐TR002369 (to OHSU).

## CONFLICT OF INTEREST STATEMENT

The authors declare no conflicts of interest.

## Data Availability

The data supporting this study's findings will be available from the corresponding authors upon reasonable request.
